# Facts and Observations on the Milk Sickness, or Sick Stomach of Tennessee, in a Letter to Dr. Samuel Brown, from Dr. Alexander M’Call

**Published:** 1830

**Authors:** 


WESTERN JOURNAL

OF THE

### MEDICAL & PHYSICAL SCIENCES.

JANUARY, FEBRUARY, & MARCH, 1830.

### ESSAYS AND CASES.

Art. I.—*Facts and Observations on the Milk Sickness, or Sick Stomach, of Tennessee;* in a letter from Dr. Alexander McCall, to Dr. Samuel Brown. 1822.

Dear Sir,—

In this essay, I shall attempt to sustain all the essential opinions and facts, contained in a thesis on milk-sickness, presented by me, to the Dean of the faculty at Philadelphia. Extracts from that thesis were published in the eighth number of the Philadelphia Journal, without my permission. The editor in his remarks, thinks the existence of such a distinct disease very improbable. I was aware when I presented that paper, it would appear in a very questionable shape, to any modern sympathies. But I also felt, that if the paper were at variance with the established axioms of the zealots in sympathy, I should view their censure of little importance. For to say the least against the wild extent, to which the recent symphathetic theory has been carried; it ought to be dispassionately revised and its more absurd parts disclaimed, before injured reason, too violently re-acting, shall at no distant time, overwhelm its beauties along with its deformities.

Medical theory is truly useful, only when its practical results are beneficial. To judge of these results, recourse must be had to the phenomena offered to clinical observers. With the view of throwing my mite into this department of our profession, I shall describe the disease, in Tennessee, usually called Milk Sickness.

Through the kindness of Dr. Sharp, I am enabled to detail several cases as they occurred in his practice. Some of the information I give, is derived from respectable citizens, who never suspected it was necessary to resort to scholastic disputations, either to prove or disprove the existence of self evident facts. The opinion of such men would be, that common sense forms a picture of passing events, which if not altered according to the taste of every froward critic, may possess lasting touches of intellectual excellence. That this belief is not without foundation, I hope can be clearly shown, by comparing the plain unvarnished statements herein detailed, with the vague and undefined hypothesis, which may be advanced in opposition.

As yet, the disease we are to consider, has not acquired a place in the systems of nosology. The 'sick stomach,' and 'puking fever,' the 'milk sickness,' and 'swamp sickness,' the 'tires,' 'slows,' and 'stiff joints,' are terms by which the complaint is designated among the commonality,—all expressing some diagnostic feature of this distressing affection.

Having premised these observations, our subject shall be treated in the manner following:

1st. Milk Sickness among inferior animals.

2. The same disease in the human family.

3. Topographical remarks.

4. Conjectures on its origin, and

5. Opinions on the structural and functional derangements, produced, constituting *ipse morbus.*

This disease originally makes its appearance amongst the inferior animals, in all its distinctive character. I will, therefore, first describe it in that state. Horned cattle, horses, deer, and sheep, are often the original subjects of the complaint. If a cow is about to be severely attacked, she exhibits signs of languor and restlessness. If she can gratify her thirst for water, it hastens the approach of the constantly occuring symptoms—universal tremors and obstinate constipation of the intestines; her head is tossed from side to side in agony, and if she remain long in one posture, the muscles will not act so as to change that position. All the muscles indeed have a peculiar rigidity. The abdominal muscles are much contracted, the body affording a thin meagre aspect, especially in the more advanced stage of the disease. If milk be now drawn it emits a peculiar odour, known to those conversant with the complaint. It is said not to froth so much as good milk,when flowing from the teat into a vessel. The cream has a greenish hue, and the butter or cheese made from it, a highly pernicious quality. If the calf sucks this milk, it trembles and often quickly falls down and dies. By abstracting the milk, the animal is partially relieved. At length her breathing becomes very laborious and offensive to the by-standers if inhaled. On dissection the stench arising from the internal parts is hardly supportable. The several apartments of the stomach, and portions of the intestines, are found in a gangrenous or sphacelous state: their contents dry, hard, and mixed with dark colored matter. Other animals are similarly affected in the violent forms of the disease. And a happy thing it were, if the disease always assumed in these animals its aggravated shape. The people would then be thrown on their guard. But it often puts on a much more insidious aspect. The first suspicion of the cow being diseased is the nausea of those, who may for several days have used her milk. Perhaps the calf is by this time also affected. If, under these circumstances, the milk be regularly drawn, the cow may exhibit no marks of disease; eating and drinking as usual. But should she be made to undergo severe exercise, the complaint will appear, in its customary form, and perhaps terminate in death, or a slow recovery of ten or twelve months. Indeed, it is stated, that animals once under the influence of this poison, never are so completely relieved, as not to feel its effects on any further violent exertion. At all stages of the complaint, ardent spirits and spirits of turpentine, have, when freely administered to cattle, had a salutary effect. After the alimentary canal is freely purged, the danger is slight, with proper care.

In Sequachee valley I saw some sheep laboring under this malady. They vomited without much apparent effort, and were also purged without having taken any medicine. They did not die. The owner, Billingsby, thought if his cattle had remained on the hills, they would not have sickened. The fatigue of driving them home and then getting water, produced the active form of the complaint.

In the dormant state of the disease the milk is no less pernicious, than in its more active form. That dogs, cats, hogs, turkeys, chickens, crows, and buzzards die by using the flesh of animals that perish under this unknown poison, the oaths of hundreds can attest.

Capt. Mills and others, informed me, that buzzards are rendered unable to fly, and that at one time on Hickerson's fork of Goose creek, sixty or seventy buzzards were found dead near the water.

Benjamin Seawell, Esq. authorised me to say, he had lost in the course of twenty years, 80 or 100 head of cattle and horses, by this poison. At one time he drove from the hills, about two miles distant from his house, fourteen steers: of which seven sickened and died at the first stream of water they crossed; two only could be driven home, and they, also, died soon afterwards. Mr. Seawell has seen dogs unable to get home after eating the flesh of dead animals, and if such dogs did not soon die, they were ever afterwards useless.—He also stated, that one of his neighbors removing his family to a distant part of the country, had six or seven horses to die on the road, with the common symptoms, although these horses were, on setting out, in apparent good health.

Mr. Elisha Henry, permits me to state on his authority, that whilst fox-hunting he found two of his dogs at a poisoned carcase. After running in the fox-chase to the first brook, and drinking, they both died.

Mrs. Britton informed me, that when her husband lay on his death bed, her milk cows ran out of the pasture by neglect. Having once had the milk sickness, she ordered her servant, before using the milk, to give some of it to a pig. The servant believing the milk good, eat some of it, and was immediately attacked; but recovered. At the sale of her husband's property, shortly afterwards, Mrs. B. purchased a mare, known to be affected by the disease, and this mare soon died. It being inconvenient to burn or bury the body, as is customary, a pen of wood was placed around; into this inclosure none but small animals could find their way. In a few days one racoon and two opossums, were found dead by the carcase.

So far as I am acquainted with milk sickness in the human family, it can be invariably traced, to the taking into the stomach the milk, butter, or flesh of animals which had been under a similar disease. There is a remarkable coincidence of symptoms presented under the operation of this peculiar poison in man and the lower animals. The violence of an attack is modified, by the quantity, and quality of the milk, butter, or flesh taken, and the time it remained on the stomach. The peculiar habits and constitution of every individual are also to be taken into consideration. Drunkards, and persons who have been several times under the influence of this poison, are liable to be effected by it. Adults are more severely attacked, in general, than children, and have a more tardy convalescence.

Not long since, I visited Goose creek, with the sole view of getting information on this subject. Unwilling that the facts connected with the disease in the human family, should rest on my own authority, alone, I applied to Dr. Sharp, an excellent practitioner of that part of the country, to give me an account of some cases that had occurred in his practice. Without a personal acquaintance, the Doctor has complied with my request. His cases are inserted at full length, and convey so correct a view of the disease, I shall not be under the necessity of saying much on that part of the subject.

DR. SHARP'S CASES.

"*Hartsville*, (*Tennessee*,) *November 24th*, 1822.

"SIR:

"I received your letters of the 2nd and 14th, requesting me to give you an account of the symptoms and treatment of the disease, commonly called milk sickness, as it exists within the bounds of my practice. I regret, that I am not able to do the subject that justice, which its importance, and the safety, and happiness of a considerable part of the inhabitants of this country demand.

However, it is, my opinion, that this disease is sui generis; and that the milk and flesh of affected animals, does produce a similar disease in other animals, using such milk and flesh; and as I am desirous that this subject should be fairly and impartially investigated by men of acknowledged talents, experience and science, I enclose a few cases—both of those successfully and unsuccessfully treated. The inhabitants of this section of country, have long been in the habit of keeping up such part of their stock, as they intended using for flesh diet, for some time before killing it. So that I am not able, to give a well marked case in the human subject, of the disease from eating the flesh of poisoned animals. I have, however, witnessed cases, which I believed were generated in that way. But I have known many horses and cattle die, quite fat, and a vast number of hogs and dogs die soon after eating their flesh, with a complaint like that under which the original animal perished.

There never has been an instance of this complaint, in any species of live stock, whilst confined to an old well cultivated pasture; nor have any persons ever been effected by using the milk or flesh of cattle thus pastured. But I have seen the disease make its appearance, in families, shortly after adding a piece of wood land to their old pastures, and disappear on separating the timbered land from the old fields.

As it respects the remote cause of this disease, however, I am not prepared to give such an opinion as should be relied on. I have searched in vain for any vegetable to which it can be ascribed; and have sometimes thought it might be a mineral poison. Nevertheless the facts which I state are substantially true, and you are at liberty to use them as you think best.

*Cases* 1 & 2. Mrs. and Mr. McNeily were taken, the one on the 2nd. and the other on the 4th day of July, 1814. The symptoms of both cases were the same; and I will give them together. A burning sensation in the stomach was first felt. It was moderate for a day or two, and not attended with any vomiting or pain. On the third day after their respective attacks, each complained of severe burning at the stomach, and vomited frequently. Obstinate constipation of the bowels attended both cases. The pulse was small, thready, and a little accelerated; and the heat of the extremities considerably below the common temperature. The tongue was tremulous and covered with white mucus. Restlessness and great anxiety prevailed in the early stage of the attack.

A constant and ungovernable thirst, was present; and when water was drunk it only served to allay the thirst for a few moments; being quickly ejected and the pain, and vomiting increased. There was no pain in the region of the liver, the spine or the head.

The stomach and bowels seemed to be the seat of the disease, and great prostration of strength soon followed. These pains with occasional hiccuping continued with unabated violence, for about twenty-four hours, from the time the vomiting commenced. The vomiting and pain then began to subside, and the hiccup became frequent. Inspiration was performed slowly and with much difficulty, and as the patients became less restless, and the anxiety abated, stupor began to prevail in the same ratio, till they became completely insensible. The pulse intermitted, the eyes beceme fixed and the palpebræ remained open; with these symptoms, death closed the scene, in about sixty hours after each began to vomit. They were freely bled, and their stomachs attempted to be composed by mucilagenous drinks and anodynes, also by the application of blisters to the region of the epigastrium. Injections of an oleaginous as well as the more drastic kind were used, repeatedly, with no good effect. Blisters were also applied to the extremities with the view of equalizing the circulation: I lost four or five patients in that neighborhood, under the same complaint, produced as I believe by using the milk of diseased cattle. There were, however, others who recovered under the plan of treatment just detailed.

*Case* 3. Miss Betsey Reid was attacked, 16th May, 1816, with burning at the stomach, attend with slight pain, she vomited almost incessantly and her thirst was constant; pulse small, thready, and frequent. She was restless, with great anxiety of countenance, in the early part of the disease, and her bowels constipated. She complained, however, of little except the burning at her stomach. She obtained momentary relief by receiving bland fluids into the stomach, but they were soon rejected. The burning at the epigastrium soon became very distressing, and great prostration of strength followed. She was bled early, but the blood would not flow freely. The arterial action seemed very languid. Mucilaginous drinks, oily purgatives, and strong cathartic enemata, were administered; but the bowels could not be evacuated; the region of the stomach, and the extremities were blistered, without producing a mitigation of the symptoms. About thirty-six hours after she began to vomit, the puking and burning suddenly ceased, but the ease she experienced for a few hours, was only the precursor of dissolution. The difficulty of breathing increasing, soon showed the delusive prospects of a recovery. The eyes became fixed and the lids open. She groaned awfully for some time, and inspiration seemed to be performed without her being sensible of it, till death closed the scene.

The duration of the disease was only three days from the time she began to puke, though she had been unwell some days before.

*Case* 4. Mr. John Reid was attacked, 20th May, 1816, with slight uneasiness at his stomach, which continued to increase till the 23rd when I prescribed for him. All the symptoms of the early stage of Miss Ried's case were present in a violent degree in his, with hiccup and cold extremities. All the remedies resorted to in her case, except bleeding, were used, without affording relief. The symptoms growing worse, I tried on the 26th the neutral salts to a considerable extent, but they were thrown from the stomach. I then commenced giving calomel, partly on account of its weight, all lighter substances being rejected from the stomach. I began by giving 10 grs. every fourth hour. This dose was continued till he took three portions. It was then increased to 20 grs. for twelve hours more; and then augmented to 40 grs. repeated at the same intervals, still using the cathartic injections. After giving the last named dose of the medicine four or five times, he began to evacuate small quantities of hardened fæces. The burning in the stomach began to subside; but he continued the last named dose one day longer, when his bowels were freely opened, and the vomiting entirely ceased, a little burning only remaining. A solution of salts, in an infusion of senna, was then substituted for the sub-mur. Hydrarg. with which a gentle catharsis was kept up a few days longer. The ptyalism was slight—the recovery speedy.

*Case* 5. Mrs. Jones was taken on the 26th of April, 1818, with slight burning at the stomach, which increased, for three days, when severe burning and some pain were felt. She often vomited, and had frequent alvine discharges, for two or three days more. Her bowels then became obstinately costive; her pulse was more open, and less corded, than is common in this complaint. The extremities were generally below the common temperature. She was restless, but as the disease advanced, she became rather stupid than otherwise. She complained of no other disagreeable symptoms.

Permit me here to observe, that in all cases which have come under my care, the matter thrown from the stomach was nearly transparent. Sometimes a little bilious, generally tasteless, and not acrid; but having a peculiar smell, a correct account of which I cannot give. I sometimes thought it faintly resembled the smell emited from the milk of cows, that had fed on young culinary vegetables, or buds of trees. At other times it has brought to my recollection, the smell of young bruised garlic. This certainly is one of the most prominent diagnostics of the the disease.

I was called on the third day of her indisposition. I bled her and commenced giving 20 grs. calomel every fourth hour, using in the mean time cathartic injections. In about twenty four hours she passed dark scybalous fæces, mixed with blackish, bloody matter. After continuing the calomel twenty four hours the salts and senna were substituted. Some days having elapsed under this treatment, she began to convalesce, and recovered speedily. I was early convinced, that her sickness was produced by using poisonous milk; and communicated my opinion to her husband, who did not think it was the case, as his milk-cow (having but one) was in perfect health. I prevailed with him to quit milking the cow. After a few days she was taken sick and died with the same symptoms, that are usual under the operation of the supposed poison.

*Case* 6. There have been a number of cases of the chronic form of this disease. I will give you that of Mr. Marshall, who was taken ill in the year 1819, with slight burning at his stomach, and occasional puking; which, at intervals, continued for several months. After a few weeks he showed evident signs of general debility; with slight and irregular exacerbations of fever. He was also afflicted with trembling and difficulty of breathing, after taking exercise. There was a general rigidity of the whole system, particularly of the joints; his bowels were often constipated, and giddiness of the head was commonly present, a symptom which, also, belongs to the acute form of this disease. His countenance was of a greenish, sallow hue, and his eyes were languid. He was always dull and inanimate in performing any thing he had to do; and rather incoherent in conversation. These symptoms harrassed him for several months. He took alkalics and saline cathartics, which scarcely ever failed to relieve him. But a recurrence of the symptoms always took place, till he refrained from the use of milk altogether; after which he was relieved by the above mentioned remedies. I am disposed to believe that this form of the disease, is only, or, at most, but little more than coeval with its cause, or in other words is kept up by the constant action of the remote cause. I have strong reason to believe, that milch cows, may labour for months together, under this disease, without showing evident signs of it. Young calves die with the usual train of symptoms, whilst their mothers appear in good health. And the flesh of such calves will communicate a similar disease to other animals feeding on it.

For the chronic form of the disease, the oily purgatives and sulphur, have long been used with much benefit; as also the pure carbonate of potash, which greatly soothes the burning sensation. You know from what has been said, that the burning and pain; vomiting and thirst; restlessness and anxiety; cold extremities and accelerated pulse; the constipated state of the bowels, and the peculiar smell before alluded to; taking into consideration the remote causes; are among the principle deagnostic of this disease. An opinion of its favorable or unfavorrble termination, may be formed, from the mildness, or violence, of the symptoms and the effects which the remedies have upon the system. One remark is worthy of being made, that I never failed of curing my patients, after their bowels had been freely and early evacuated.

I have selected these cases, of old date, merely because I had memoranda of them for my own use, as they occured in the course of my practice. There have been many such cases of more recent date. However, the citizens have been, for three or four years past, much more careful of their stock, and consequently the cases are fewer in number.

The mode of treatment, now followed by me, is such as stated in Mrs. Jones' case, with the addition of blistering the epigastric region, and varying the doses of medicine to suit circumstances. I find under this plan of practice, I can save six out of nine of my patients, whereas by the former method I lost nearly all. Bland drinks are of some service; but since we see the bowels partake so much of the disease of the stomach, it is plain that purgatives are chiefly to be relied on.

The duration of the complaint in its acute form is various; it often runs its course in less than five days, but sometimes extends to ten or more. It occurs at any season of the year, but is most common during the months of March, April, May, September, October, and November.

I am sir with respect,

M. D. L. F. SHARP."

"DR. McCALL."

To give several cases I had prepared, which are similar to those detailed by Dr. Sharp, would be useless, even if the limits of this paper would permit. I have the histories of a number of cases which occured in the state of North Carolina, between the years 1779 and 1800; but they differ in no material point from those of Dr. Sharp. I must not, however, omit to mention on the authority of Maj. Martin, the fact of a childs dying in consequence of sucking its diseased mother. The lady resided near Goose creek, and contracted the disease by using the milk of a cow labouring under a similar affection. The child survived the mother several days.

A case somewhat analogous is related in Coxe's Medical Museum, 3rd volume; by Dr. S. T. Barstow, May 27, 1806.

"Some time in the summer of 1801, the wife of Mr. Alfred Bremin, in the town of Baintrim, Luzern county, Pennsylvania, was bitten by a rattlesnake. She was then in fourth month of pregnancy. After some considerable degree of the common consequences of such an accident had occured; she at length recovered. At the full time of delivery she was safely put to bed. The child was apparently healthy; but immediately after allowing it to suck, it assumed the hues of a rattlesnake, swelled very much and soon died. She then procured a puppy which died in two days of the same symptoms, and in succession one puppy and three lambs shared the same fate. Another puppy was procured which discovered but little of the symptoms and did not die. In 1803 she had another child, and no disagreeable symptoms resulted from the use of her milk."

I once saw a child having its skin much discoloured with yellow and dark spots. Its parents stated, that about one year previous, it was bitten by a rattle snake. That a discolouration similar to that it then had, occured soon after it was bitten. That the spots had disappeared during the winter, and they supposed it to be restored to health. But when the season approached in which it was bitten, the skin again became discoloured, and its extremities swelled. The child died.

I will conclude this part of my subject by giving the certificates of John Miller and his son, of Goose creek in regard to the milk sickness.

"I have been in the habit," says the former, "of keeping up my stock for twenty years past. About four years ago my sheep were turned out of the pasture, and soon after seven or eight died. Not supposing they were poisoned, I did not burn their carcases, and my hogs eat of them. These hogs died and were destroyed, except one which I did not find in the lot. This body was found to be picked by the chickens which chickens, as I believe caused milk sickness in myself, my wife, and three children, by using them as diet. My reason for believing so, is, that we had the true milk sickness, and had eat no other fresh meat; and my son who eat none of the chickens, or soup, was not sick. My cattle had not been out of the old lot, and he as well as the rest of us used their milk. What, however, acted as convincing proof to my mind, was, that so long as we used chicken soup as diet, in our sickness, we all grew worse: till suspecting the fowls for being the cause, we abstained from using them. My wife died; and I still feel the complaint after taking exercise.

John Miller."

"Buzzards eating the diseased flesh often die and some of them lose the power of flying for some days. One of my dogs having partaken of diseased beef, run after a rabit across the field, and then fell down and died; another dog died in the same way, whilst in pursuit of a hog.

John Miller, Jun."

Whether or not Mr. Miller be right in his conjecture, of the cause of sickness in his family, one thing it seems is ascertained, that the poison can in three animals successfully generate disease, by only using the flesh of each one as it dies.

I will now offer some topographical remarks, on some of the districts, in which the disease is met with. The Goose creek tract is on the north side of the Cumberland river. At the distance of 10 or 15 miles from the river, a chain of highlands runs, separating the waters of the Cumberland, from those of Barren river in Kentucky. From this ridge appending hills set off at near right angles; those running south, dividing the branches of Goose creek towards their sources. Along these ridges animals contract the disease. The chain running between the upper and middle forks of Goose creek, rises in some places from two to three hundred feet above the level of the river. Of these hills the "Mill stone knob," is the most remarkable. From its summit a country beautifully variegated with hills and dales, in a high state of cultivation presents itself. The mill stone quarry whence it takes its name, is about two thirds of the distance from the base to the summit. Viewing the face of this quarry where it will be seen that only a few feet of rich mould, covers a flaky bed of slate, which last lies on the mill stone rock. When heat is applied to these mill stones, an oily substance exudes, in combination, with some sulphur. The general face of the country presents no appearances worthy of notice, more than the adjacient lands.

Without giving a particular description of them, I will mention several other places where the disease occurs. I have seen the disease with many of its peculiarities, in Stokes county, North Carolina, in the low lands of Yadkin river. It was there called the "River sickness," and could always be eradicated by cultivating the soil, as was amply proven on Kirby's and Poindexter's farms. This was within a mile of the place of my nativity; and these facts are among my earliest recollections. It was thought to be produced by what is commonly called wild parsnip roots, as many of those roots were exposed on the surface of the ground, by hogs turning up the soil. A species of spider with its webs, adhering to the vegetables, was also supposed by some, to be the remote cause of the milk sickness.

On the little Yadken river, where there were iron works, it was by others, ascribed to the mineral exhalations raised by heat and subsiding on the vegetation. In the same state, it occurs in Burk county, and I believe in Guilford county. Also, in some parts of South Carolina and Georgia. I think, in some part of Dr. Rush's works, it is mentioned, that a disease had occured in one of the Southern states, from the use of vitiated milk. Linneus, in his tour through Lapland, discovered a plant which had been deleterious to horned cattle. But I have not his journal before me.

To return to our disease, it occurs both in the mountainous and flat lands of Kentucky; in the plains of Indiana, of Mississippi, of Missouri, of Illinois, Michigan, and especially in Ohio. In the state of Tennessee, it is not confined to Goose creek. In the year 1821, the Legislature of the state, passed an act, to have fences made round certain coves of the Cumberland mountain, in Franklin county: to prevent (as it reads) animals eating an unknown vegetable, thereby imparting to their milk and flesh qualities highly deleterious. The provisions of this act, specify the duties of the overseers of such fences &c. I am informed that the fence is now in good repair, twelve or fifteen miles along the base of the mountain. The disease is also met with in Smith county, and in Bedford county; on the waters of the French broad river, in East Tennessee; and near some of the branches of the rivers Emery, and Clynch. It is met with in Sequachee valley, Bledsoe county, where as I before observed, I have witnessed distressing cases of milk sickness. This valley is about seventy miles in length, and generally from five to fifteen miles in breadth, lying between the Cumberland mountains on one side, and the walnut ridge, its auxiliary chain, on the other side. From these two lofty mountains, this valley in the summer months, presents a delightful prospect of numerous and well improved farms—a little world in calm and lowly seclusion.

The atmosphere on these broad based and elevated mountains, is singularly pure and serene, and during the spring and summer months, the morning traveller arriving at the summits of these mountains, will, on viewing the foggy valley below, be impressed with the idea of an extended and fluctuating lake.

Passing, sometime since, through this valley, in company with my father, he informed me, that at an early period of the settlement of this country, when narrow paths only directed the footsteps of the anxious traveller, he unexpectedly came to the brink of what he supposed was a lake, as far as his eyes could see on either hand. The sun was shining, and the imitative waves rolling beneath in uncommon grandeur, made him conclude that he was far out of his way; till the green tree tops, projecting through, disclosed the deception. In going down to the valley, the wild, broken precipices of each mountain, present a bold and striking picture of those dreadful convulsions, which, in times long past, effected their separation and formed this garden of the hills. Yet even in this delightful valley, animal life is annoyed by the disease under consideration. Not a neighbor, friend, or relative, falls under its mysterious power, without an anxious excitement to know something more of this enemy to our peace and health. Who that has any humanity will longer withhold his aid in searching out its hidden cause? But inquiries to be efficient, should be properly directed and applied. By proper investigation I have no doubt the remote cause of milk sickness can be detected. Although, perhaps, like many causes doing injury to the animal system, its detection might be of little practical benefit.

It will be readily supposed, that various conjectures have been made on the probable cause of milk sickness. If not instructive, it may be amusing to take a brief view of some of these notions. Many suppose it is the effect of one of the mineral poisons. Mountainous districts where minerals abound are, sometimes, the fruitful source of milk sickness. Many mineral substances are known to have peculiar effects on some of the secretory functions. It is also thought that arsenic in a vaporised state might be the poison that subsides on vegetables, and this opinion is said to be supported by the fact, or opinion that animals only contract disease whilst dew is on the herbage. Inferring perhaps from this, that the sun has the effect of dissipating such effluvia, or that, in the absence of light and heat, vegetable life has the power of decomposing the atmosphere in contact with its leaves, by which a poisonous substance is produced on the surface of the leaves, which, could not have been generated in a vitiated air without vegetable existence, nor by the vegetative powers, had not an impure atmosphere been presented. Were it a mineral exhalation, the state of the atmosphere would have some influence over it. Rain and snow would arrest it, and to say the least, it would require a higher degree of temperature than that of our winter days, to vaporise any mineral. If it be a mineral exhalation why does it not appear in all places where there are large quantities of iron, sulphur, copper, arsenic and lead, in different states of combination? Why would not the exhalation be wafted across a river, or why will it not arise in a piece of ground where the old vegetation has been destroyed? In grass lots there is sufficiency of herbage for it to fall on, if such exhalation exists. From all the facts given in Dr. Sharp's paper, and from my own observations, I must for the present come to the conclusion, that some vegetable is the remote cause. It is not impossible that two or more vegetables taken into the stomach may jointly produce effects, which singly used, could not have occured. I design making a regular set of experiments with the flesh and milk of poisoned animals. Much information might perhaps be got, by examining the stomachs of animals on the first appearance of the complaint. The substance from the human stomach should also be examined; and the milk supposed to be poisoned ought to be accurately analysed.

*Editorial Note.*—The Author of the forgoing paper has several pages on the modus operandi, of the supposed poison which produce Milk sickness, and on the pathological condition of the system in that malady; they are ingenious speculations, but as they embrace no experiments nor autopsic examinations, and as our object is to record facts, we shall omit their publication.

Within the last ten years, many papers have been published in the Journals of America, on this disease; which, still remains of a doubtful character, in its ætiology, nosological relations, and therapeutics. We have not, ourselves, had an opportunity of witnessing the disease, though according to the reports of some of our professional friends, residing not far from Cincinnati, in various directions, it is with them not an uncommon malady. It seems indeed to belong to the *country*, and to be endemic in particular districts only.

Our first knowledge of this complaint dates back as far as the year 1809. In 1810, the Editor of this Journal appended to a small pamphlet, entitled, "Notices Concerning Cincinnati," (a few copies of which were printed for distribution,) a short, but as far as we know the first, notice of this malady. As the facts which it sets forth, make a part of the history of the disease, and are not within the reach of the Profession; we have deemed it not improper to transfer them to the pages of the Western Journal.

"New Disease.

"In the spring of 1809, Dr. Barbee, of Virginia, on returning from a visit to the Madriver county in this state, gave me some information concerning a new and formidable disease which had appeared among the settlers of that tract. Since that time, I have been able to collect serveral additional facts respecting it, from different persons, more especially Mr. William Snodgrass, and Mr. John M'Kag, two intelligent and respectable inhabitants of that country, who have several times experienced the disease in their persons and families. A summary of the whole, is here given, that physicians may determine how far it deserves the appellation of a new disease.

It almost invariably commences with general weakness and lassitude, which increase in the most gradual manner. About the same time, or soon after, a dull pain, or rather soreness, begins to affect the calves of the legs, occasionally extending up to the thighs. The appetite becomes rather impaired, and in some cases nearly suspended; sensations of a disagreeable kind affecting the stomach; upon taking a little food, however, a greater disposition for it is generated, and more agreeable feelings are introduced throughout the whole system. Intestinal constipation, in this, as in the subsequent periods of the disease, exists in a very high degree. A strong propensity to sleep occurs, and according to Dr. Barbee, the pulse is "full, frequent, round, and *somewhat* tense, but regular." During this stage, exercise of any kind is highly ditrimental, and if persisted in, soon inducing loathing and nausea at the stomach. If the patient repose, upon first experiencing these symptoms, they generally cease, and he is allowed a longer exemption from the *vomiting* that awaits him. Sooner or later, however, that symptom almost invariably succeeds the predisposition we have described, and either proves fatal in one, two, three or more days, or leaves the patient in a most exhausted state, from which he recovers only to sustain, at no distant period, a repetition of the same attack.

The matter ejected is sometimes bilious, but much oftener sour, and so acrid, that its action on the throat, in one case, (which proved fatal) was likened to that of boiling water. Towards the close of mortal cases, it is occasionally very dark colored, so that it has been compared to that very convenient and fashionable object of simlitude —coffee grounds. At this time the intestinal constipation is very great. Mr. Snodgrass knew one patient in whom it continued for nine days, throughout which he took no food whatever, and vomited during six of them. After such an attack, the propensity of it is destroyed, and an uncommon degree of watchfulness is produced. The patient remains languid, and his face and person generally become rather tumid. His skin is cool, palish, and frequently affected with clamminess. He has a disagreeable burning sensation in his stomach, and hot eructations are very troublesome. The thirst is considerable. The breath is peculiarly disgusting, even loathsome. The appetite is generally poor; and the inclination to costivness remains. These symptoms often continue for several months, during which the patient experiences frequent returns of the vomiting. But at length, more especially upon the approach of winter, they gradually wear away, leaving the patient considerably worse than they found him, and liable to a fresh attack the ensuing summer.

Nothing like *regular* periodical exascerbations is observable in this disease; no chillines occurs; the color of the skin and eyes does not deviate widely from that of health, and gives no striking indication of bile; there is no pain in the region of the liver, nor in the shoulder; it does not terminate in dropsy; nor are there any symptoms which bespeak it a disguised or anomalous intermittent. It however prevails (though not exclusively) in aguish situations, and intermitting diseases are thought to have declined since its appearance.

It affects all ages, conditions, and both sexes, indiscriminately; except probably very young children. They, however, are not wholly exempt from it. Emigrants are not peculiarly liable to it. It was first observed in the summer of 1806, and is thought annually to extend its geographical range, and to become more intense. It sometimes commences in July or before, but oftener in August, and continues till the approach of winter, when it generally but not always subsides.

The cure of this disease seems hitherto to have been left chiefly to the people, who have not yet discovered any certain method. Purging was a remedy that naturally suggested itself; and by some it has been thought very serviceable, more especially when effected by aloes; but others assert that they have frequently known a cathartic to increase the vomiting, and therefore rely more on enemata. All agree however, that the intestinal obstructions are to be overcome; and that the less the means made use of, affect the stomach, the better. Vomits evidently do harm. Blisters to the gasiric region are considered the most efficient remedy. Tonics have been used, but no great benefit appeared to arise from them. Wine and salted meats, however, have appeared to do good, and are relished beyond any thing else. Indeed, eating a little frequently, whether an inclination exist or not, has been found a good palliative. It relieves the stomach from the gnawing which so perpetually exists. Alkaline lye has been used in one case: it gave some tempoary relief, but not more than almost any other substance which might be received into the stomach. Bleeding has occusionally been resorted to, but with doubtful advantage. Ardent spirits appears to render the disease worse: it is not, however, much sought after, all inclination for it, generally being destroyed. Tea and coffee, also, with several other articles of diet, which were agreeable before the disease, are in many cases disliked for a long time after.

The disease is unequivocally observed to affect four domestic animals; the horse, the cow, the sheep, and the dog. It is often fatal to the two former; but not so fatal to the latter. It as frequently attacks horses in the winter as summer, and sometimes them in 24 hours.

It prevails chiefly in the neighborhood of Staunton on the Great Miami, and in the country south of Madriver, between Dayton and Springfield. In those tracts, ponds and marshes occasionally occur, more especially in the former. The soil and water are calcarous. The timber generally oak."

